# Simulation Study on Temperature and Stress Fields in Mg-Gd-Y-Zn-Zr Alloy during CMT Additive Manufacturing Process

**DOI:** 10.3390/ma17051199

**Published:** 2024-03-05

**Authors:** Mingkun Zhao, Zhanyong Zhao, Wenbo Du, Peikang Bai, Zhiquan Huang

**Affiliations:** 1 School of Materials Science and Engineering, North University of China, Taiyuan 030051, China; sz202103064@st.nuc.edu.cn; 2 National Key Laboratory for Remanufacturing, Academy of Army Armored Forces, Beijing 100072, China; dwbneu@163.com; 3 School of Materials Science and Engineering, Taiyuan University of Science and Technology, Taiyuan 030602, China; 4 School of Mechanical Engineering, Taiyuan University of Science and Technology, Taiyuan 030602, China; huangzhipeng607@163.com

**Keywords:** simulation, residual stress, cold metal transfer, combined heat source, magnesium rare earth

## Abstract

A new heat source combination, consisting of a uniform body heat source and a tilted double ellipsoidal heat source, has been developed for cold metal transfer (CMT) wire-arc additive manufacturing of Mg-Gd-Y-Zn-Zr alloy. Simulations were conducted to analyze the temperature field and stress distribution during the process. The optimal combination of feeding speed and welding speed was found to be 8 m/min and 8 mm/s, respectively, resulting in the lowest thermal accumulation and residual stress. Z-axis residual stress was identified as the main component of residual stress. Electron Backscatter Diffraction (EBSD) testing showed weak texture strength, and Kernel Average Misorientation (KAM) analysis revealed that the 1st layer had the highest residual stress, while the 11th layer had higher residual stress than the 6th layer. Microhardness in the 1st, 11th, and 6th layers varies due to residual stress impacts on dislocation density. Higher residual stress increases dislocation density, raising microhardness in components. The experimental results were highly consistent with the simulated results.

## 1. Introduction

Cold metal transfer welding (CMT) is an extremely promising manufacturing technology. It precisely controls the metal transfer by accurately controlling the delivery process of the welding wire. At the same time, it avoids unnecessary heat input through alternating cooling and heating with high current and low voltage. Through the above methods, efficient, spatter-free, and low-heat-input additive manufacturing is achieved [1,2,3]. Due to its high additive efficiency and low residual stress characteristics, it has shown a wide range of application prospects in the aerospace and automotive industries. Hu [4] and others successfully used CMT to manufacture AZ31 magnesium alloy by adjusting the process parameters. Wang [5] and others investigated the influence of voltage and current on the morphology of CMT wire-arc additive AZ31 magnesium alloy. The study found that the strong pulsing CMT heat input process can deposit wide and shallow welds, which is suitable for the parameters of CMT wire-arc additive AZ31 magnesium alloy.

Magnesium alloys have the significant effect of reducing weight in the automotive and aerospace industries due to their low density and high specific strength [6,7,8,9,10,11,12,13]. In recent years, rare earth magnesium alloys have received increasing attention due to their high performance at both room and high temperatures [14,15,16,17]. Li et al. [18] used computer-aided cooling curve analysis technology to study the solidification pathways and structures of Mg-Gd-Y-Zr alloys with different Gd and Y concentrations. The results showed that the structure of the alloy was mainly composed of α-Mg matrix and Mg_24_RE_5_ phase. Meier et al. [19] obtained the phase diagram of Mg-10Gd-xY-yZn alloys with different Y and Zn ratios through experiments and CALPHAD phase diagram calculations and determined the phases and fractions produced after casting and solid solution treatment. The results showed that the phases found at different Y and Zn concentrations were mainly Mg matrix and 14H (LPSO) phase. The volume fraction of the LPSO phase was significantly increased after solid solution treatment.

Due to the high thermal expansion coefficient of magnesium alloy and the extremely high heat input frequency of CMT wire-arc additive, the additive region is susceptible to high residual stress after experiencing extremely complex thermal cycles [20]. The high residual stress can cause cracking and even geometric failure of the workpiece. Therefore, the study of the effect of process parameters on the residual stress of CMT wire-arc additive magnesium alloy is crucial for promoting the widespread application of CMT wire-arc additive manufacturing.

Finite element analysis is an increasingly accurate predictive means used to determine the thermal behavior of welding and additive manufacturing processes and to determine stress–strain in additive manufacturing processes [21,22]. Zhao et al. [21] studied the influence of deposition direction on residual stress in single-bead multi-layer additive manufacturing processes using finite element models. The study found that reverse deposition had the best effect. Li et al. [20] established a combined heat source to simulate the impact of path strategies on residual stress in a laser-CMT hybrid additive manufacturing process. The study found that the Segmental Reciprocating Motion (SRM) strategy resulted in the smallest distortion during the additive manufacturing process.

In the past, most of the stress field simulations in additive manufacturing focused on residual stress simulations of single-bead and multi-layer without overlap [23,24,25,26,27,28]. The influence of overlap on temperature and stress fields is rarely considered. The morphology of the weld has a great influence on the simulation results of temperature and stress fields. Simple geometric models cannot reveal the complex thermal stress evolution process in the additive region. Especially in wire-arc additive manufacturing, the weld size and the size of overlapping areas that have been heavily remelted are large, and this area has undergone extremely complex thermal cycles, which has a great impact on the simulation results. Therefore, it is necessary to establish a numerical simulation model that is closer to the real weld morphology, which is of great significance to improve the simulation accuracy [29,30,31,32].

In the past, most simulation models for wire-arc additive manufacturing used double ellipsoidal heat sources. However, due to the inherent defects of double ellipsoidal heat sources, it is difficult to completely melt the arc-shaped weld during the simulation process, and it is also impossible to accurately simulate the morphology of the melt pool. This will cause a large deviation between the simulation results of the temperature and stress fields and the actual production. Therefore, it is necessary to establish a new heat source model for wire-arc additive manufacturing. This paper will establish a new combined heat source composed of a uniform body heat source and an inclined double ellipsoidal body heat source and use this heat source to simulate the temperature and stress fields of rare earth magnesium alloy during the CMT wire-arc additive manufacturing process under different process parameters. By comparing the simulation results, suitable process parameters can be found and used to add magnesium alloy.

## 2. Experimental Procedure

The wire-arc additive equipment used in this experiment is the Fronius monolithic welding machine. The CMT (1101) MnE21 magnesium welding parameter combination is used. The welding speed is 6–8mm/s, the wire feeding speed is 8–10 m/min, and the interlayer cooling time is 100 s. The working parameters corresponding to different wire feeding speeds are shown in Table 1. The substrate and the wire used for welding are Mg-Gd-Y-Zn-Zr alloys, and their material composition proportions are shown in Table 2. The wire diameter used for wire-arc additive manufacturing is 1.2 mm, and the substrate size is 200 mm × 50 mm × 40 mm. A unidirectional scanning path is used during the additive process.

All microstructure characterization was carried out parallel to the YOZ plane because this direction contains the fish-scale-like morphology of the melt pool. For the EBSD sample preparation, the sample was mechanically polished and then etched for 15 s in a solution of nitric acid and ethanol (5 g nitric acid, 95 g ethanol) (Linyi Lugang Chemical Group Co., Ltd., Linyi, China). EBSD experiments were conducted on a Sigma300 scanning electron microscope (Zeiss, Jena, Germany) with a scanning step of 0.25 μm and a magnification of 500×. The XRD equipment used is the Rigaku SmartLab (Rigaku Corporation, Tokyo, Japan). Hardness tests were performed five times in each region, and the average value was taken. The hardness testing equipment used is the HVS-1000 (Shanghai Yanrun Optomechanical Technology Co., Ltd., Shanghai, China). Compression performance tests were conducted three times in each region, and the best performance curve was selected for use. The compression test was performed using an Instron 3382 (Instron, Norwood, MA, USA).

## 3. Establishment of Simulation Model

### 3.1. Finite Element Model

**Figure 1:**
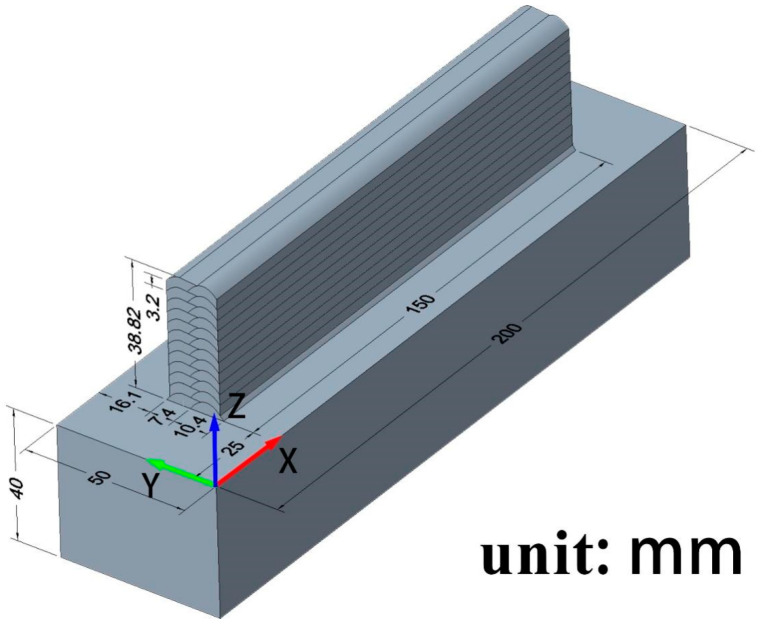
Model established using Creo for a wire feeding speed of 8 m/min.

The dimensions of the model are shown in Figure 1. The model was created using Creo 8.0 and then imported into ANSYS 2022 for modification.

**Figure 2:**
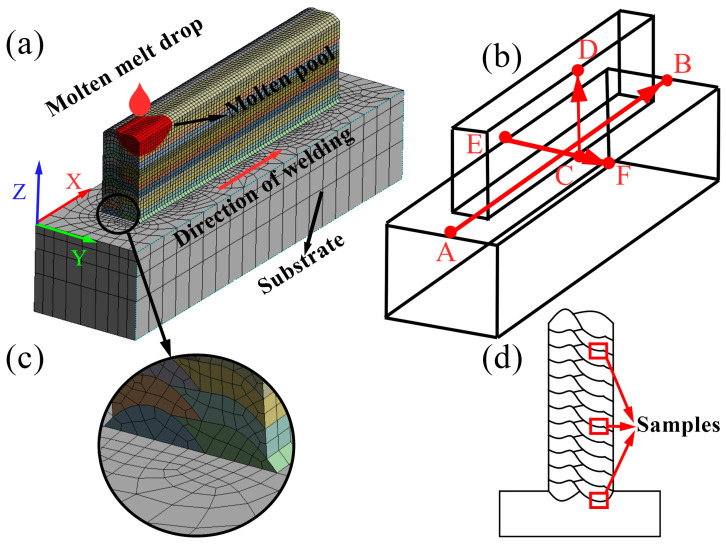
Schematic diagram of model establishment: (**a**) schematic diagram of CMT process; (**b**) schematic diagram of stress field data extracted along the path; (**c**) details of finite element meshing; (**d**) schematic diagram of sampling position.

The model was meshed using ANSYS. Sparse mesh cannot guarantee the accuracy of the calculation, and overly dense mesh greatly increases the computation [33]. The mesh size was set between 1 and 3 mm, which corresponds to different welding speeds. To better depict the morphology of the additive zone and obtain more precise results, the Solid186 element was used for the mesh, as shown in Figure 2a. To reduce computational workload, denser mesh was used near the additive region to increase computation accuracy, while coarser mesh was used away from the additive region to reduce computational workload, as shown in Figure 2c. Figure 2a shows the scanning strategy of the model [21]. The additive region is divided into 12 layers, each layer with two paths, and the researched point and sampling position are both located at x = 100 mm. Figure 2b shows the extraction route of residual stress simulation data. Figure 2d displays the selected positions of samples for the 1st, 6th, and 11th layers. The birth and death element technique was used to simulate the additive process of each layer.

### 3.2. Combined Heat Source Model

**Figure 3:**
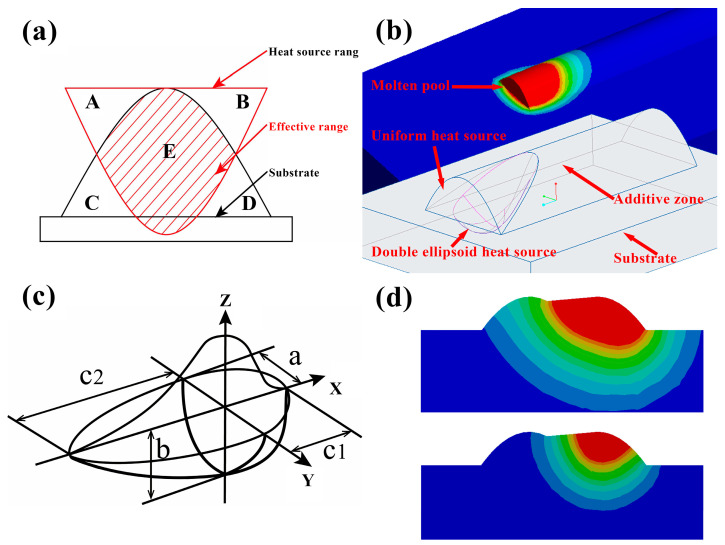
Heat source diagram: (**a**) double ellipsoid heat source loading diagram in arc weld bead; (**b**) combined heat source loading diagram in arc weld bead; (**c**) traditional double ellipsoid heat source diagram; (**d**) comparison of the melt pool morphology between the novel heat source and the double ellipsoid heat source under the same process parameters.

Due to the consideration of the heat distribution inside the molten pool, the double ellipsoid heat source makes the simulation results close to reality and is widely used in additive manufacturing simulation [34]. However, when the traditional double ellipsoid heat source is loaded on the arc bead, the heat source is not fully loaded, as shown in Figure 3a. The AB region of the heat source is not applied to the additive region, and the CD part of the additive region is not loaded with a heat source. Therefore, the heating of the CD region can only rely on heat conduction, resulting in the region not being heated to the melting temperature. If the traditional heat source is used for CMT arc additive manufacturing simulation, the results of the temperature field and stress field will deviate far from reality. To resolve the aforementioned issues, this paper combines a uniform heat source with an inclined double ellipsoidal heat source, as shown in Figure 3b. The homogeneous body heat source is applied to the upper part of the additive area. Since the homogeneous body heat source can be loaded in any shape of the entity, it can be loaded in the CD area. Under the uniform heat source is the inclined double ellipsoid heat source. On the one hand, it can better simulate the depth of the molten pool. On the other hand, it can make the temperature in the molten pool close to the Gaussian distribution to make up for the deficiency of the uniform body heat source so that the temperature field simulation is more accurate. The two heat sources are loaded at the same time but need to be set separately because of different energies and shapes. Figure 3d displays the morphology of the melt pool created by the novel combined heat source at the top and that formed by the double ellipsoid heat source at the bottom. It is evident from the figure that there is a significant difference in the melt pool morphology under the same process parameters and identical double ellipsoid dimensions. The traditional double ellipsoid heat source, due to its inability to be fully applied across the entire weld track, results in a melt pool morphology that deviates significantly from reality. This discrepancy can lead to severe errors in the simulation of temperature and stress fields.

Since two heat sources are considered in this paper, the total arc power will consist of two parts:

(1) $$P=\eta UI=P_{a}+P_{b}$$

In the formula, η is the arc thermal efficiency, U and I are the voltage and current of the arc, respectively. $P_{a}$ represents the power of the homogeneous body heat source, and $P_{b}$ represents the power of the double ellipsoid heat source. The heat flux density of the homogeneous heat source can be expressed as follows:

(2) $$q_{a}=\frac{P_{a}}{V_{a}}$$

where $V_{a}$ is the volume of the activation unit above the double ellipsoid heat source in a time step increment ΔT, as shown in Figure 3b.

The traditional double ellipsoid heat source model is divided into two parts, and the model is shown in Figure 3c. The heat flux density expression of a double ellipsoid heat source is as follows [35]:

(3) $$q_{b1}=\frac{6\sqrt{3}f_{1}P_{b}}{\pi abc_{1}\sqrt{\pi }}\exp\left(-3\frac{x^{2}}{c_{1}^{2}}\right)\exp\left(-3\frac{y^{2}}{a^{2}}\right)\exp\left(-3\frac{z^{2}}{b^{2}}\right)$$

(4) $$q_{b2}=\frac{6\sqrt{3}f_{2}P_{b}}{\pi abc_{2}\sqrt{\pi }}\exp\left(-3\frac{x^{2}}{c_{2}^{2}}\right)\exp\left(-3\frac{y^{2}}{a^{2}}\right)\exp\left(-3\frac{z^{2}}{b^{2}}\right)$$

(5) $$f_{1}+f_{2}=2$$

where $f_{1}$ and $f_{2}$ are the heat source distribution coefficients of the first half and the second half of the heat source. a, b, $c_{1}$, and $c_{2}$ are the size of the double ellipsoid heat source.

Because the double ellipsoid heat source used in this paper tilts a certain angle around the y-axis, a local coordinate system with a certain tilt angle relative to the global coordinate system is defined in Ansys Parametric Design Language (APDL), and the heat flux of each node in the double ellipsoid heat source is calculated in the local coordinate system so as to achieve the purpose of tilting the heat source.

### 3.3. Thermodynamic Analysis

The transient energy equation used to describe the overall volume heat transfer process in the wire-arc additive process is as follows:

(6) $$\dot{H}=-\nabla \cdot q+\dot{Q}+{\dot{D}}_{mech}$$

In the formula, $\dot{H}$ is the enthalpy rate, q is the heat flux, $\dot{Q}$ is the heat source, and ${\dot{D}}_{mech}$ represents the heat engine dissipation. The heat flux q can be calculated by the Fourier law:

(7) q=−k∇t

where k is the thermal conductivity of the material at different temperatures and ∇t is the variation in temperature.

### 3.4. Stress Analysis

The momentum balance equation of quasi-static mechanical analysis includes residual stress and deformation, which can be written as follows:

(8) $$\rho \ddot{u}+\mathrm{div}\left(\sigma \right)+b=0$$

In the formula, ρ is the density of the material, σ is the Cauchy stress tensor, u is the displacement vector, $\ddot{u}$ is the second derivative of time, and b is the volume force vector of the model. According to Hooke’s law, the stress tensor is related to the elastic strain:

(9) $$\sigma =D:\varepsilon^{e}$$

where D is the elastic stiffness matrix determined by Young’s modulus (E) and Poisson’s ratio (v).

The total strain increment can be composed of the following parts:

(10) $$\Delta \varepsilon =\Delta \varepsilon^{e}+\Delta \varepsilon^{p}+\Delta \varepsilon^{t}+\Delta \varepsilon^{v}$$

**Figure 4:**
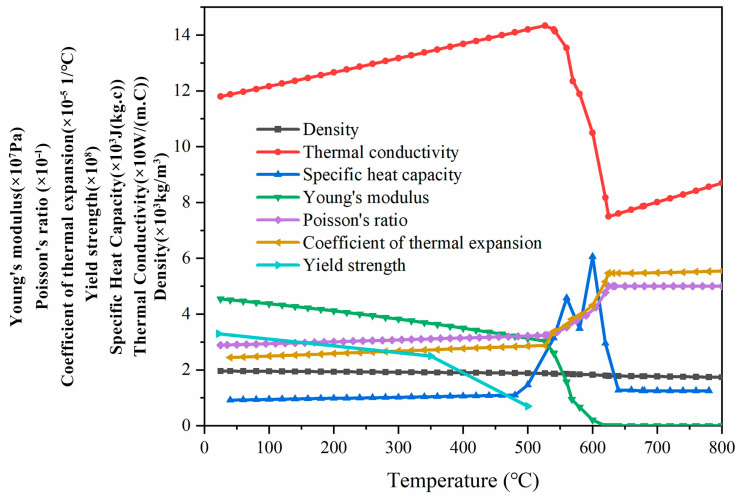
The thermophysical parameters of Mg-9.2Gd-3.2Y-2Zn-0.4Zr alloy calculated using JMatPro during cooling from 800 °C to room temperature.

In the formula, $\varepsilon^{e}$, $\varepsilon^{p},$ and $\varepsilon^{t}$ are elastic strain, plastic strain, and thermal strain, respectively. $\varepsilon^{v}$ is the volume strain caused by phase transformation and creep, which is not considered in this model. The calculation formula of thermal strain $\Delta \varepsilon^{t}$ is as follows:

(11) $$\Delta \varepsilon^{t}=A\Delta T$$

(12) $$A^{T}=\beta \left[111000\right]$$

where ΔT is the temperature increment and *β* is the temperature-dependent thermal expansion coefficient, as shown in Figure 4.

The elastic strain increment component $\Delta \varepsilon_{ij}^{e}$ can be described by the stress component $\sigma_{ij}$.

(13) $$\Delta \varepsilon_{ij}^{e}=\frac{1+v}{E}\Delta \sigma_{ij}-\frac{v}{E}\Delta \sigma_{kk}\delta_{ij}$$

In the formula, $\delta_{ij}$ is Kronecker δ. Plastic strain is caused by yield and strain hardening, $\sigma_{ij}$ is deviatoric stress, and $\sigma_{kk}$ is hydrostatic pressure. In this model, assuming that the plastic zone is isotropic hardening, the plastic strain increment according to the normality rule can be calculated as follows:

(14) $$\Delta \varepsilon^{p}=\frac{1}{3}\Delta \varepsilon_{p}I+\Delta \varepsilon_{q}n$$

where n is the flow direction and I is the unit matrix. p and q are two mutually perpendicular directions. $\varepsilon_{p}$ and $\varepsilon_{q}$ can be calculated by the following equations:

(15) $$\phi \left(p,q,H^{\alpha }\right)=0$$

(16) $$\Delta \varepsilon_{p}\frac{\partial \phi }{\partial q}+\Delta \varepsilon_{q}\frac{\partial \phi }{\partial p}=0$$

In the formula, $H^{\alpha }$ is a set of hardening parameters, and p, q, and $H^{\alpha }$ are defined by the following equations [36,37]:

(17) $$p=p^{el}+K\Delta \varepsilon_{P}$$

(18) $$q=q^{el}+3G\Delta \varepsilon_{P}$$

(19) $$\Delta H^{\alpha }=h^{\alpha }\left(\Delta \varepsilon_{p},\Delta \varepsilon_{q},p,q,H^{\beta }\right)$$

where $H^{\beta }$ is the hardening modulus and G and K are the shear modulus and bulk modulus.

### 3.5. The Initial and Boundary Conditions of Simulation

The initial conditions in the simulation can be described as follows:

(20) $$T{\left.\left(x,y,z,t\right)\right|}_{t=0}=T_{0}$$

where $T_{0}$ is the ambient temperature, and the ambient temperature is 25 °C.

The boundary conditions in the simulation can be defined as follows:

(21) $$k\frac{\partial T}{\partial n}=q_{S}-q_{conv}-q_{radi}$$

where n is the normal vector, $q_{S}$ is the input heat flux, $q_{conv}$ is the thermal convection, and $q_{radi}$ is the thermal radiation. Thermal convection and thermal radiation can be obtained by the following equations:

(22) $$q_{conv}=h(T-T_{0})$$

(23) $$q_{radi}=\sigma \varepsilon \left(T^{4}-T_{0}^{4}\right)$$

where h is the convective heat transfer coefficient, σ is the Stefan–Boltzmann constant, which is 5.67 × 10^−8^ W/m^2^ K^4^, and ε is the emissivity.

### 3.6. Thermal Physical Parameters of Materials

The thermal physical parameters of the materials in this paper are calculated by JmatPro 7.0 software. JMatPro can simulate the phase diagram and physical properties of alloy materials. The substrate and wire used in this paper are Mg-9.2Gd-3.2Y-2Zn-0.4Zr. The thermal physical parameters of the material required for the simulation of the temperature field and stress field are shown in Figure 4, and the stress–strain curve of the material is referred to [38].

In the course of this work, the following assumptions are made:

1. The thermophysical properties of the additive area and the substrate are isotropic;
2. There is no spatter during the forming process;
3. The metal does not evaporate during the forming process;
4. The size of the additive area remains constant at the same wire feeding speed;
5. Under different process parameters, the height of the additive area is consistent, and the thickness of each layer is identical.

Based on existing reports, the microstructure of cast and additive-manufactured rare earth magnesium alloys is fully equiaxed, and thus, this paper does not consider the influence of material property anisotropy. In the CMT forming process, due to the extremely low heat input, occurrences of spattering are rare; consequently, the variations in heat input caused by spattering and changes in deposited volume are negligible. Since data extraction for simulation requires path tracking, the changes in the model size must not be excessive, as this would complicate the comparison of simulation data. Overall, the model has been partially simplified for the sake of simulation efficiency and to facilitate subsequent comparisons. While this may affect the precision of the simulation to some extent, it does not alter the trend of the conclusions. The experiments later in the text also validate the accuracy of the simulation.

## 4. Simulation Conclusions and Experimental Results

### 4.1. Temperature History

**Figure 5:**
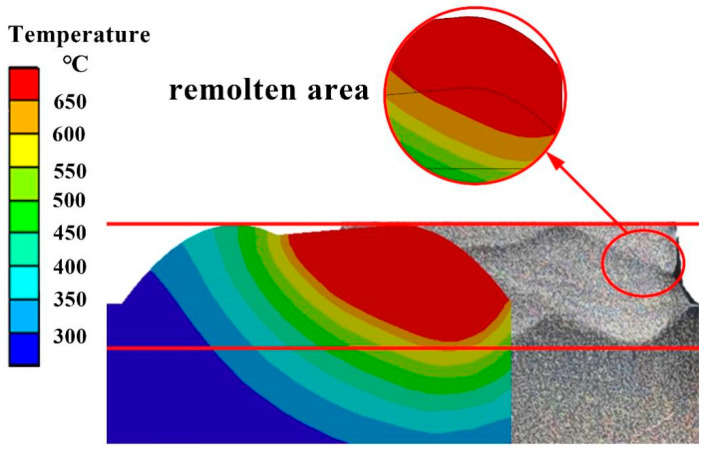
Comparison of simulated molten pool morphology and actual molten pool morphology.

Firstly, the heat source was calibrated and tested. Then, after depositing two layers with two passes using a rare earth magnesium alloy, the specimen was etched for 15 s in a nitric acid ethanol solution (10 g nitric acid mixed with 80 g ethanol). The heat source is calibrated by comparing the actual weld pool size with the simulated weld pool size, as shown in Figure 5. Upon employing a novel heat source, the morphology and dimensions of the melt pool can be precisely predicted, and the simulation of the remelted region also achieves a high degree of accuracy. It is proved that the new combined heat source is very suitable for CMT wire-arc additive manufacturing simulation.

**Figure 6:**
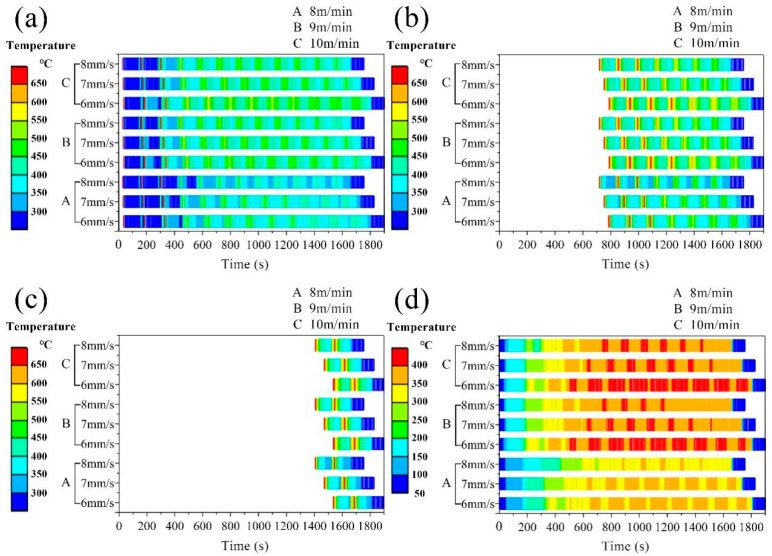
Thermal history simulation results under different process parameters, where A, B, and C represent wire feeding speeds, and within the parentheses of A, B, and C are the welding speeds: (**a**–**c**) represent the thermal history of the nodes at the center of the first, sixth, and eleventh layers of the second weld track, respectively; (**d**) denotes the minimum temperature during the additive manufacturing process.

The thermal history of the midpoints of the 1st, 6th, and 11th layers was extracted for analysis, and the thermal behavior of different layers of additive manufacturing was studied. Figure 6a–c shows the simulated thermal history of different layer midpoints at different wire feeding speeds and welding speeds. Figure 6d reflects heat accumulation through the history of minimum temperature during the additive process. Different colors in the figure represent different temperature ranges, and the projection of each color in the X-axis direction represents the duration of the temperature range. In Figure 6a–c, the temperature of 650 °C and above is set to red, indicating that the point is completely melted. It can be seen from the diagram that with the increase in welding speed, the liquid phase existence time of the molten pool gradually decreases, the cooling speed of the additive region increases, and the heat accumulation degree gradually decreases.

From the B and C processes in Figure 6b, it can be seen that the faster the welding speed is, the fewer times the sixth layer of the additive region is remelted, which means that the faster the welding speed is, the shallower the molten pool depth is. With the increase in wire feeding speed, the existence time of the molten pool liquid phase increases, the cooling rate of the additive region decreases, and the degree of heat accumulation gradually increases. 

From Figure 6b, it can be seen that with the increase in wire feeding speed, the number of times that the sixth layer of the additive region is remelted increases, which means that the faster the wire feeding speed, the deeper the molten pool depth. With the increase in the number of additive layers, the time of liquid phase existence gradually increases, the cooling rate of the additive region decreases, and the heat accumulation degree increases. 

Compared with Figure 6a,b, it can be seen that with the increase in wire feeding speed, the number of remelting in the additive region increases. Comparing the thermal history of different layers, it can be seen that the sixth layer is remelted more times than the first layer, which means that the depth of the molten pool increases with the increase in the number of layers. At the same time, the higher the number of additive layers, the longer the liquid phase life, the lower the cooling rate in the additive region, and the higher the heat accumulation degree. Fundamentally, variations in thermal history attributable to disparate processing parameters stem from differential levels of heat accumulation. An uptick in heat retention prompts an expansion in the dimensions of the molten pool, a reduction in the rate at which it cools, and a lessened temperature gradient, given a steady power input. These factors collectively give rise to the previously described occurrences. 

Figure 6d illustrates the thermal history of the lowest temperature in the system during the additive manufacturing process under various process parameters. The minimum temperature of the system at the commencement of the formation of a new layer is regarded as an indicator of thermal accumulation. The thermal input during the additive manufacturing process results in an increase in the part’s temperature, and due to the brief interlayer cooling period, it is unable to cool down to room temperature. Consequently, this leads to an elevated initial temperature of the part when depositing a subsequent layer compared to the initial temperature of the previous layer, thereby resulting in thermal accumulation. It is found that the degree of heat accumulation increases with the increase in feeding speed. With the increase in welding speed, the degree of heat accumulation decreases. Heat accumulation increases first, then decreases, and finally, the region is stable in the process of additive manufacturing. Heat accumulation will reduce the cooling rate of the molten pool, resulting in coarse grains, thereby reducing the strength of the parts after additive manufacturing. Therefore, in order to obtain a refined structure, a wire feeding speed of 8 m/min and a welding speed of 8 mm/s with the lowest degree of heat accumulation should be selected to form rare earth magnesium alloy parts.

**Figure 7:**
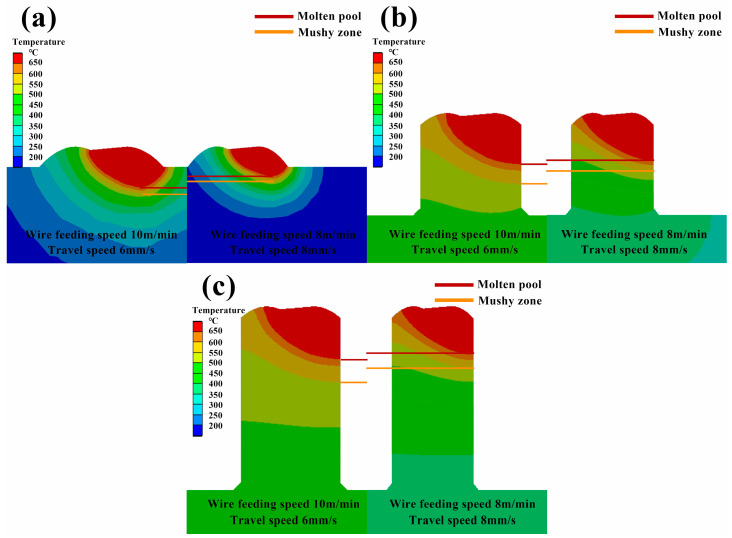
Comparison of simulated melt pools using the process parameters with the maximum and minimum heat input: (**a**–**c**) depict comparative simulation results for the melt pools in the first, sixth, and eleventh layers, respectively.

From the perspective of heat input, the voltage and current are constant at the same wire feeding speed, so the lower the welding speed, the higher the total heat input, resulting in an increase in heat accumulation. As the wire feeding speed increases, the voltage current increases, so the total heat input during the welding process increases, resulting in an increase in heat accumulation. Therefore, in the range of the above process parameters, the total heat input of the process parameters with a wire feeding speed of 8 m/min and a welding speed of 8 mm/s is the lowest, and the total heat input of the process parameters with a wire feeding speed of 10 m/min and a welding speed of 6 mm/s is the largest. Figure 7 illustrates the temperature field distribution on the longitudinal sections of the 1st, 6th, and 11th layers under process parameters with the highest and lowest total heat input. The comparison reveals that with the increase in the additive manufacturing layers, there is an increase in heat accumulation, which consequently leads to a greater depth of the molten pool and an expansion of the mushy zone. The higher the heat input, the deeper the molten pool, the deeper the mushy zone, and the larger the heat-affected zone.

### 4.2. Residual Stress Distribution

**Figure 8:**
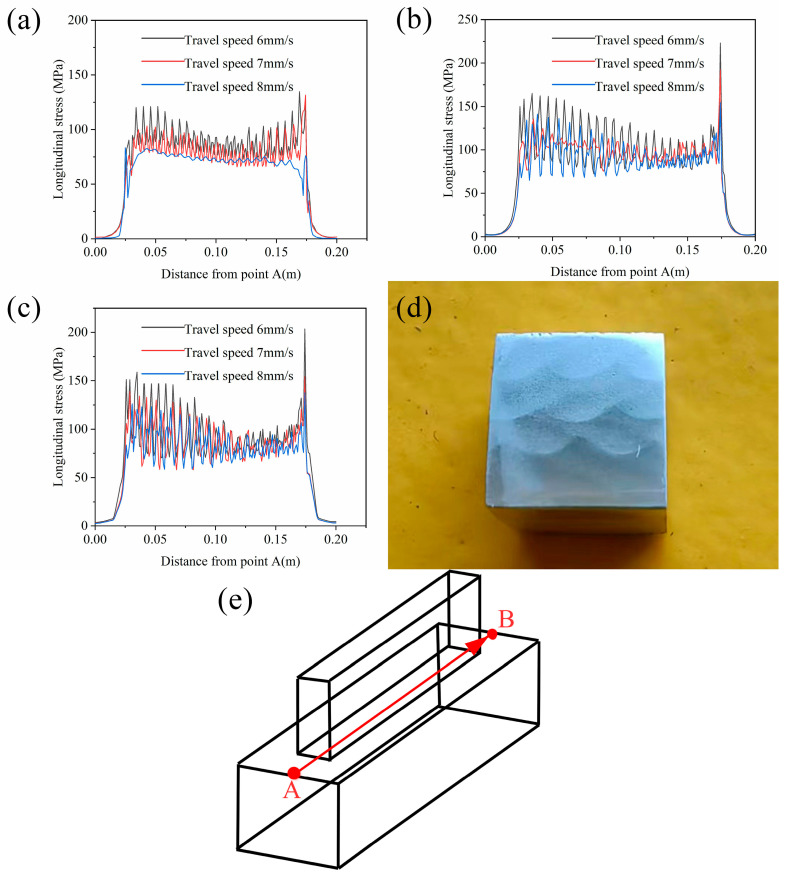
Residual stress distribution along path A, B under varying process parameters: (**a**–**c**) display the residual stress distributions for wire feeding speeds of 8 m/min, 9 m/min, and 10 m/min, respectively; (**d**) illustrates the actual cross-sectional morphology of the melt pool; (**e**) schematic diagram of stress field data extracted along the path.

Figure 8 shows the residual stress distribution along the AB line under different process parameters. It is found that the faster the wire feeding speed, the higher the residual stress, and the faster the welding speed, the lower the residual stress. At the same time, the closer to the initial arc point, the higher the residual stress is. This is because the substrate temperature is lower at the beginning of the additive manufacturing, and the cooling rate of the molten pool is faster, so the residual stress is higher. As the welding proceeds, the substrate is heated, which reduces the cooling rate of the molten pool and reduces the stress. The stress at the arc-extinguishing point is significantly increased because the arc-extinguishing point is only heated and melted once, without experiencing stress unloading after remelting. The closer to the arc starting point, the greater the stress fluctuation is. This is because, during the CMT wire-arc additive manufacturing process, the discontinuity of the heat source loading leads to the discontinuity of the molten pool, as shown in Figure 8d, which makes the residual stress fluctuate. As the first layer of additive continues, the lower substrate is heated, resulting in an increase in the size of the molten pool and an increase in the continuity of the molten pool, resulting in a decrease in the degree of stress fluctuation.

**Figure 9:**
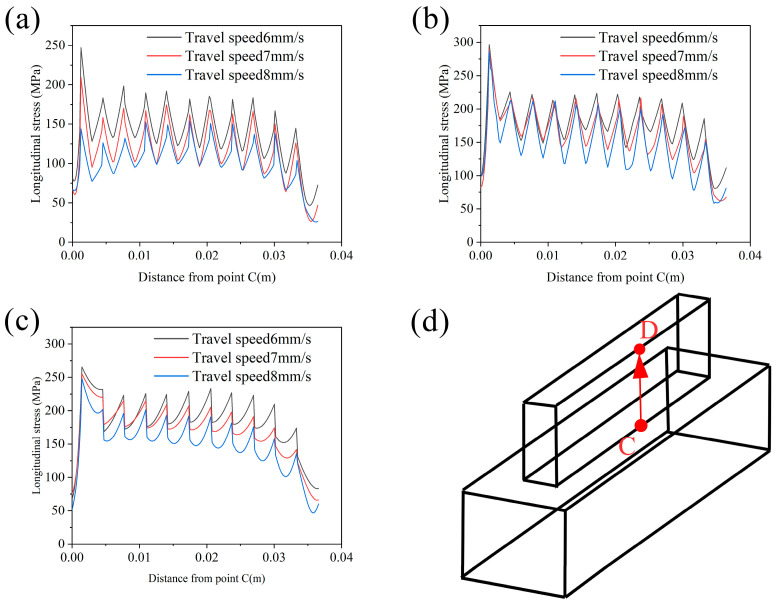
Distribution of residual stress along path C, D under different process parameters: (**a**–**c**) illustrate the distributions of residual stress at wire feeding speeds of 8 m/min, 9 m/min, and 10 m/min, respectively; (**d**) schematic diagram of stress field data extracted along the path.

Figure 9 shows the residual stress distribution along the CD line under different process parameters. By comparison, it can be found that the residual stress increases with the increase in wire feeding speed and decreases with the increase in welding speed. The residual stress of the first layer is the largest because the first layer has almost no heat accumulation, and the higher cooling rate leads to higher residual stress. The residual stress value fluctuates between layers. This is because, except for the last layer, each layer of the additive region has experienced the remelting of the upper pass. When these beads are remelted for the last time, the molten pool cannot completely melt the pass. Therefore, the degree of stress release in the same pass is different, which leads to the fluctuation of residual stress along the CD line. From the figure, the residual stress between the layers does not decrease with the remelting, but the residual stress of the last two layers decreases significantly. This will be explained below.

**Figure 10:**
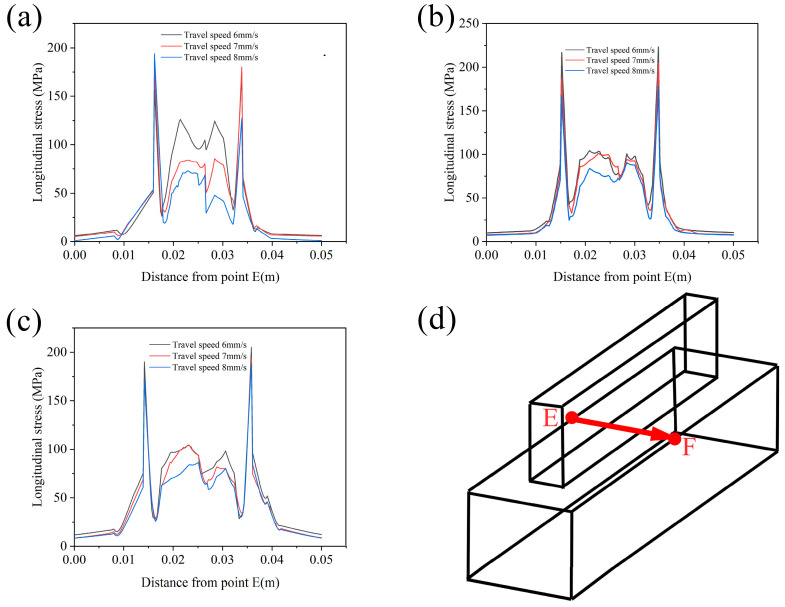
Distribution of residual stress along path E, F under disparate process parameters: (**a**–**c**) correspond to the residual stress distributions at wire feeding speeds of 8 m/min, 9 m/min, and 10 m/min, respectively; (**d**) schematic diagram of stress field data extracted along the path.

Figure 10 shows the residual stress distribution along the EF line under different process parameters. By comparison, it can be found that the residual stress increases with the increase in wire feeding speed. With the increase in welding speed, the residual stress decreases. The stress value is larger at the edge of the beads and the substrate connection. This is because the strain caused by the shrinkage of the Y direction during the cooling of the additive region accumulates to the edge of the additive, resulting in a larger residual stress. The stress near the lap area of the second pass is significantly reduced, which is due to the second pass remelting the edge of the first pass and the connection of the substrate, resulting in stress unloading.

Through the stress field simulation, it is found that the lower the wire feeding speed, the lower the residual stress, and the lower the welding speed, the higher the residual stress. As a rule, reduced heat input correlates with diminished residual stress. Therefore, when using 1.2 mm diameter Mg-9Gd-3Y-2.2Zn-0.5Zr wire, within the above process parameters, when CMT wire-arc additive manufacturing is performed on the same material substrate, the residual stress is the lowest at an 8 m/min wire feeding speed and an 8 mm/s welding speed. Therefore, from the perspective of reducing residual stress, this process parameter should be used for additive manufacturing.

**Figure 11:**
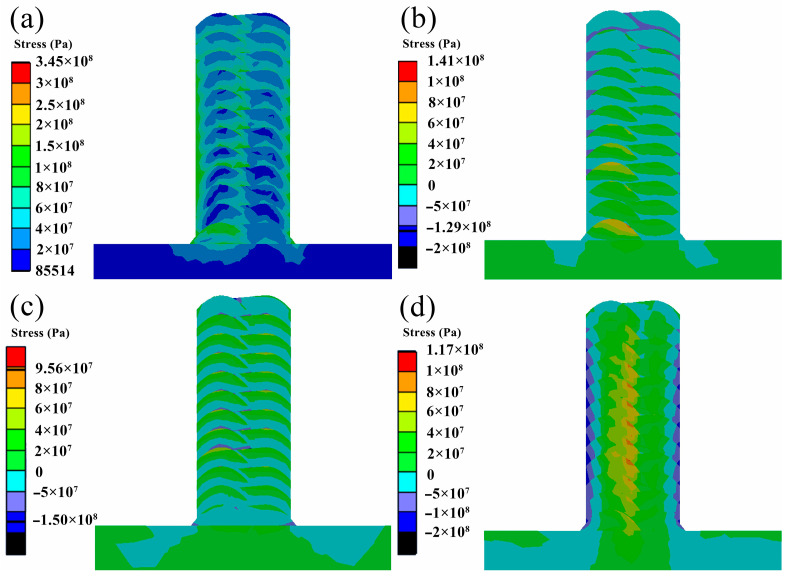
Residual stress distribution of longitudinal section under the process parameters of a wire feeding speed of 8 m/min and a welding speed of 8 mm/s: (**a**) equivalent stress distribution; (**b**) residual stress distribution in X direction; (**c**) residual stress distribution in Y direction; (**d**) residual stress distribution in Z direction.

In order to explore the distribution law of residual stress after additive manufacturing, the simulation results of the longitudinal section stress field under the lowest residual stress process parameters are shown in Figure 11. Figure 11a shows the equivalent stress distribution of the longitudinal section, and the residual stress is mainly concentrated at the edge of the additive region and near the lap area. The residual stress in the central area of each bead is low, and the residual stress increases with the increase in the layer height. The first layer has the highest residual stress because it has the fastest cooling rate. 

Figure 11b shows the residual stress distribution in the X direction. It can be seen from the figure that the upper part of the beads of each layer mainly bears tensile stress, and the lower part mainly bears compressive stress. This is because the upper additive region remelts a part of the lower layer during the additive process. The remelted area generates tensile stress in the X direction during the solidification shrinkage process, so the lower part of the remelted area is subjected to compressive stress in the X direction. When the additive is completed, the cooling rate of the top layer of the additive region is lower than that of the bottom layer due to the influence of heat accumulation. Therefore, the bottom layer shrinks before the top layer, resulting in more tensile stress on the bottom layer and more compressive stress on the top layer. Therefore, the tensile stress decreases with the increase in the number of layers, and the compressive stress increases with the increase in the number of layers. 

Figure 11c shows the residual stress distribution in the Y direction. It can be seen from the figure that the tensile stress and compressive stress interval distribution in the additive region are similar to the above. The residual stress of the first layer is mainly compressive stress. This is because the size of the substrate in the Y direction is much larger than that of the additive region, so greater deformation occurs during the cooling shrinkage process, resulting in greater compressive stress in the first layer of the additive region. The residual stress distribution of the other layers does not change with the layer height, which is due to the consistent size of each layer in the Y direction. 

Figure 11d shows the residual stress distribution in the Z direction. It can be seen from the figure that the central part of the additive region is mainly subjected to tensile stress, and the edge of the additive region is mainly subjected to compressive stress. The tensile stress is mainly concentrated near the lap area, and the compressive stress is mainly concentrated on the surface near the additive region. Since the heat is mainly dissipated from the outer surface of the additive region in the form of radiation and convection during cooling, the cooling rate of the outer surface of the additive region is higher than that of the center of the additive region. During cooling, the outer surface first cools and shrinks, while the central region does not shrink due to cooling because the temperature is higher than the outer surface, so the outer surface is subjected to tensile stress. The center of the additive area is subjected to compressive stress, and plastic deformation occurs. Subsequently, the center of the additive region is cooled and contracted, and its internal stress is changed from compressive stress to tensile stress. At the same time, the tensile stress near the outer surface is changed from tensile stress to compressive stress. This process is repeated until cooled to room temperature. The reason why the tensile stress in the Z direction is mainly concentrated in the overlapping area of the additive center is that the position is located at the center of the additive region, and the longer the above process, the more the tensile stress is concentrated here. The outer surface of the additive region also undergoes more of the above process, leading to compressive stress concentration. 

By analyzing the residual stress distribution in different directions of the section, it can be seen that the residual stress near the outer surface of the additive region and the center of the additive region is mainly the compressive stress in the Z direction. Therefore, the wider the additive region, the greater the residual stress in the Z direction. This also explains that the residual stress along the CD above does not change significantly with the change in layer height because the residual stress in the Z direction is related to the distance from the outer surface, while the CD line is on the outer surface, so the residual stress does not change with the layer height. The lower residual stress in the central region of each layer of the section is caused by the lower residual stress in the X and Y directions. With the increase in layer height, the residual stress in the central area of each layer increases, which is caused by the increase in compressive stress in the X direction.

Through the stress field analysis, it is found that the residual stress in the Z direction is dominant. Figure 7 reveals that the higher the heat input, the deeper the molten pool in the Z direction, the larger the mushy area, and the larger the heat-affected area. As a result, in the process of solidification and cooling, the degree of shrinkage deformation in the Z direction of the additive region is greater, which leads to an increase in residual stress in the Z direction and, finally, increases the overall residual stress.

### 4.3. Experimental Result

**Figure 12:**
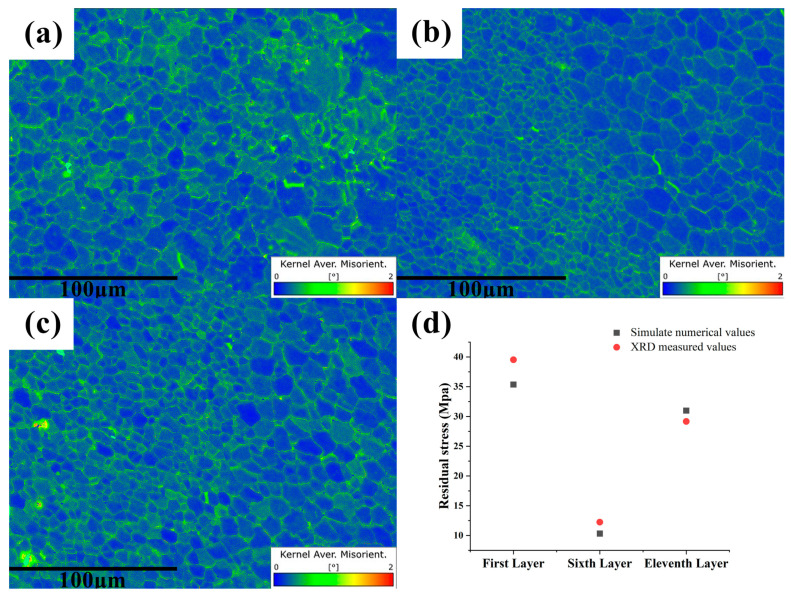
Distribution of the KAM in the longitudinal section under the process parameters of an 8 m/min wire feeding speed and an 8 mm/s welding speed: (**a**–**c**) represent the KAM distribution at the first, sixth, and eleventh layers, respectively; (**d**) the comparison of residual stress simulation values with experimental values for each layer.

Using the best process parameters, a 12-layer rare earth magnesium alloy part was fabricated. Samples were taken from the 1st, 6th, and 11th layers for EBSD (Electron Backscatter Diffraction) analysis. Additionally, the residual stresses at the center of the weld beads for each layer were measured using X-ray diffraction and compared with simulated data. Figure 12 uses the Kernel Average Misorientation (KAM) diagram to represent the average orientation difference between two adjacent points [39,40,41]. The KAM diagram shows that the residual stress of the 1st layer is the largest, and the residual stress of the 11th layer is greater than that of the 6th layer. The residual stress in the fine-grain zone of the 6th and 11th layers is greater than that in the coarse-grain zone. The residual stress of the first layer is close to that of the substrate. The distribution of the mean orientation difference in the grain and the scanning map show that the mean orientation difference is larger at the grain boundary, which indicates that the residual stress causes the dislocation to move to the grain boundary and be pinned by the second phase at the grain boundary. Figure 12d compares the actual residual stresses measured by X-ray diffraction with the simulated residual stress values. The numerical agreement is suitable, validating the accuracy of the simulation. There is a slight discrepancy between the simulated data and the actual measurements, which can be attributed to the fact that the residual stresses measured by XRD represent an average over a plane, whereas the internal residual stress distribution in the formed part is non-uniform and complex. Therefore, the measured residual stress values may differ from the simulated results [42,43].

**Figure 13:**
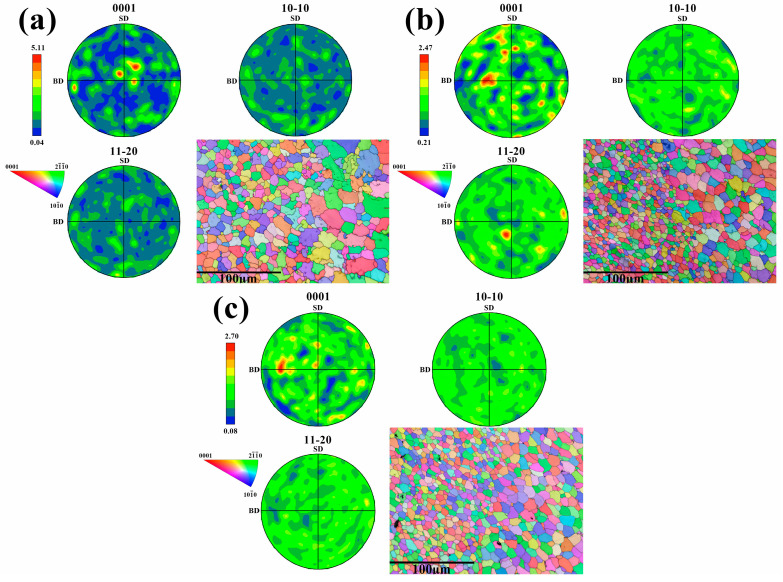
Electron Backscatter Diffraction (EBSD) maps under the process parameters with a wire feeding speed of 8 m/min and a welding speed of 8 mm/s: (**a**–**c**) respectively correspond to the pole figures and Inverse Pole Figure (IPF) maps of the first, sixth, and eleventh layers.

Figure 13 shows the pole figures and IPF figures of the (0001) plane, (10–10) plane, and (11–20) plane in the 1st layer, the 6th layer, and the 11th layer. The additive region of the alloy is composed of equiaxed crystals, and the crystals have no obvious orientation. The texture strength of each layer is low, and its maximum value is only 5.11 in the first layer (0001) plane. Therefore, the Mg-Gd-Y-Zn-Zr alloy manufactured by additive manufacturing is uniform and isotropic. The different stress distribution of each layer has no significant effect on the crystal orientation.

**Figure 14:**
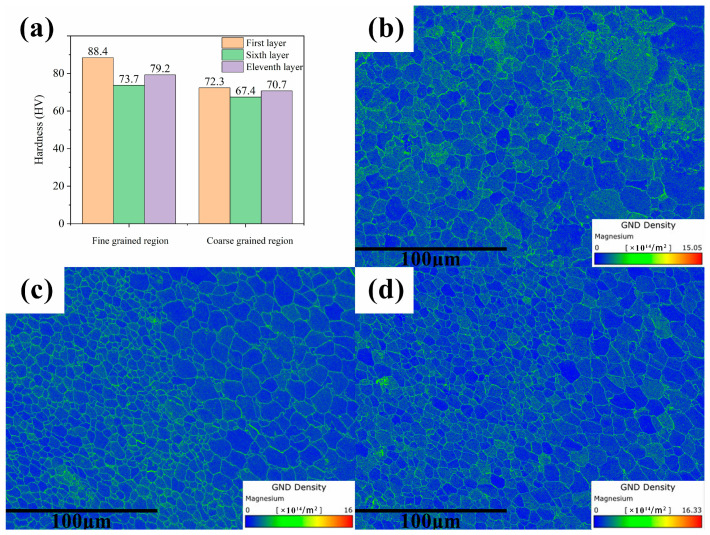
Hardness magnitude and Grain Boundary Density (GND) distribution under the process parameters with a wire feeding speed of 8 m/min and a welding speed of 8 mm/s: (**a**) presents the hardness values for the coarse-grained and fine-grained zones in each layer; (**b**–**d**) depict the GND distribution for the first, sixth, and eleventh layers, respectively.

Figure 14 illustrates the microhardness test results for the coarse- and fine-grain regions in the 1st, 6th, and 11th layers of the specimens, as well as the geometrically necessary dislocation (GND) results for each layer. Upon examination, the microhardness of the fine-grain regions in all layers was found to be significantly higher than that of the coarse-grain regions. Comparing the microhardness among the layers, the first layer exhibited the highest microhardness, while the sixth layer showed the lowest. By combining the hardness test results with stress field simulation outcomes, it was observed that regions with higher residual stresses exhibit increased hardness. Comparing the dislocation densities among the layers revealed that the 1st layer has a higher dislocation density than the 11th layer, which is higher than the 6th layer. Layers with higher dislocation densities exhibit greater microhardness. It is well known that the hardness of a crystal is closely related to its dislocation density [44,45,46]. As a large number of dislocations exist within a crystal, they interfere with each other, generating pinning and entanglement between dislocations, which impedes dislocation slip and consequently augments the hardness of the crystal. Therefore, it can be concluded that the residual stresses after the CMT wire-arc additive manufacturing process significantly alter the hardness of Mg-Gd-Y-Zn-Zr alloy. Higher residual stresses in a component lead to a larger dislocation density within the grains, thereby increasing its microhardness.

**Figure 15:**
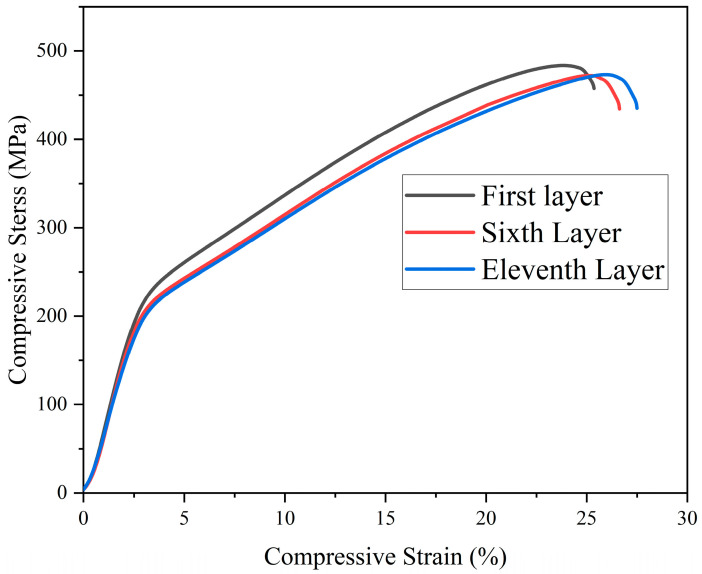
Engineering stress–strain curves for compressed specimens.

Figure 15 shows the compressive engineering stress–strain curves of each layer, where the first layer had the highest yield strength and compressive strength, which was due to its largest residual stress and larger plastic deformation that caused the generation of a large number of dislocations in the grains during the deformation process. In the microstructure, grain size and dislocation pinning both have an impact on the material’s physical properties. Although a larger grain size is disadvantageous for yield strength and compressive strength, higher-density dislocation pinning can still enhance yield strength and compressive strength [47,48]. This explains why the first layer has higher yield and compressive strengths than other layers despite having a larger grain size; it is the result of a trade-off between these two influencing factors. However, dislocation pinning cannot improve the material’s plasticity, and the larger grain size makes it difficult for grains to roll during deformation, which results in poorer plasticity in the first layer. The reason why the 11th layer had higher yield strength and compressive strength than the 6th layer was also related to the dislocation density. Therefore, higher residual stress can improve the yield strength and compressive strength of Mg-Gd-Y-Zn-Zr alloy by producing larger plastic deformation during the cooling process.

## 5. Conclusions

In this paper, a new combined heat source is established to simulate the temperature field and stress field of the CMT wire-arc additive manufacturing process. By comparing the thermal history and residual stress under different process parameters, the most suitable process parameters for wire-arc additive manufacturing of Mg-Gd-Y-Zn-Zr alloy were found. The stress distribution was detected by EBSD after forming magnesium alloy parts with the best process parameters. The simulation results are consistent with the experimental results. The effects of different process parameters on thermal history and residual stress were analyzed. At the same time, the thermal history and residual stress of different layers with the same process parameters were analyzed. The main conclusions are as follows:

(1) With the increase in heat input, heat accumulation increases. As a result, the liquid phase life of the molten pool increases, the size of the molten pool increases, the mushy area becomes larger, the heat-affected area becomes larger, and the cooling rate decreases. The greater the wire feeding speed, the greater the heat input, and the lower the welding speed, the lower the heat input. The process parameters with the lowest heat input and the lowest heat accumulation are a wire feeding speed of 8 m/min and a welding speed of 8 mm/s.
(2) The residual stress is mainly concentrated on the outer wall of the additive region and the center of the additive region. The residual stress in the Z direction plays a major role. The stress concentration at the arc-extinguishing point of the substrate is because the number of thermal cycles at the arc-extinguishing point is less than that in other areas, and there is no sufficient release stress to cause stress concentration. Through simulation, it is found that the greater the wire feeding speed, the greater the residual stress. The lower the welding speed, the greater the residual stress. The process parameters with the lowest residual stress are a wire feeding speed of 8 m/min and a welding speed of 8 mm/s.
(3) The grain texture of each layer after additive manufacturing is weak, and the residual stress has no obvious effect on the grain orientation. KAM analysis shows that the residual stress of the 1st layer is the highest, and the residual stress of the 11th layer is higher than that of the 6th layer. This is consistent with the simulation results.
(4) The microhardness of the 1st layer was higher than that of the 11th layer and the 6th layer, which was attributed to the residual stress that affected the dislocation density and, consequently, the microhardness of each layer. The higher dislocation density also enhanced the yield strength and compressive strength of the material. The larger residual stress led to the higher dislocation density, which eventually resulted in the increased microhardness and mechanical properties of the part.
(5) The experimental results show that the new combined heat source is very suitable for the simulation of the temperature field and stress field of Mg-Gd-Y-Zn-Zr alloy fabricated by CMT wire-arc additive manufacturing.

## Figures and Tables

**Table 1:** CMT operating parameters with different wire feeding speeds.

Wire Feeding Speed (m/min)	Voltage (V)	Current (A)
8	11.3	87
9	11.7	93
10	12	100

**Table 2:** Proportions of each element in rare earth magnesium alloy.

Material	Mg	Gd	Y	Zn	Zr
Content (wt%)	Bal.	9.2	3.2	2	0.4

## Data Availability

Data are contained within the article.
